# Genome-wide identification, new classification, expression analysis and screening of drought & heat resistance related candidates in the RING zinc finger gene family of bread wheat (*Triticum aestivum* L.)

**DOI:** 10.1186/s12864-022-08905-x

**Published:** 2022-10-07

**Authors:** Yongliang Li, Pai Qin, Aolong Sun, Wenjun Xiao, Fenglin Chen, Yang He, Keyao Yu, You Li, Meng Zhang, Xinhong Guo

**Affiliations:** College of Biology, Hunan University, Changsha, 410082 China

**Keywords:** *Triticum aestivum*, RING zinc finger, Genome-wide analysis, Expression patterns

## Abstract

**Background:**

RING (Really Interesting New Gene) zinc finger (RING-zf) proteins belong to an important subclass of zinc fingers superfamily, which play versatile roles during various developmental stages and in abiotic stress responses. Based on the conserved cysteine and histidine residues, the RING-zf domains are classified into RING-HC (C3HC4), RING-H2 (C3H2C3), RING-v, RING-D, RING-S/T, RING-G, and RING-C2. However, little is known about the function of the RING-zfs of wheat.

**Results:**

In this study, 129 (93.5%) of 138 members were found in nucleus, indicating TaRING-zf were primarily engaged in the degradation of transcription factors and other nuclear-localized proteins. 138 TaRING-zf domains can be divided into four canonical or modified types (RING-H2, RING-HC, RING-D, and RING-M). The RING-M was newly identified in *T. aestivum*, and might represent the intermediate other states between RING-zf domain and other modified domains. The consensus sequence of the RING-M domain can be described as M-X_2_-R-X_14_-Cys-X_1_-H-X_2_-Cys-X_2_-Cys-X_10_-Cys-X_2_-Cys. Further interspecies collinearity analyses showed that *TaRING-zfs* were more closely related to the genes in *Poaceae*. According to the public transcriptome data, most of the *TaRING-zfs* were expressed at different 15 stages of plant growth, development, and some of them exhibited specific responses to drought/heat stress. Moreover, 4 RING-HC (*TraesCS2A02G526800.1*, *TraesCS4A02G290600.1*, *TraesCS4B02G023600.1* and *TraesCS4D02G021200.1*) and 2 RING-H2 (*TraesCS3A02G288900.1* and *TraesCS4A02G174600.1*) were significantly expressed at different development stages and under drought stress. These findings provide valuable reference data for further study of their physiological functions in wheat varieties.

**Conclusions:**

Taken together, the characterization and classifications of the TaRING-zf family were extensively studied and some new features about it were revealed. This study could provide some valuable targets for further studies on their functions in growth and development, and abiotic stress responses in wheat.

**Supplementary Information:**

The online version contains supplementary material available at 10.1186/s12864-022-08905-x.

## Background

The RING (Really Interesting New Gene) ZFPs are one of types of E3 Ubiquitin (Ub) ligases according to their different catalytic domains. The RING domain is a zinc finger (ZF) type protein structural region that contains 40–60 amino acid residues. Their consensus sequence can be described as Cys-X_2_-Cys-X_(9–39)_-Cys-X_(1–3)_-His-X_(2–3)_-Cys/His-X_2_-Cys-X_(4–48)_-Cys-X_2_-Cys. The RING-zf domain is a Cys-rich domain with 8 metal ligand positions and four pairs of Cys/His residues that coordinate binding of two zinc ions in an across-brace structure [1, 2]. However, unlike proteins with canonical ZF domains that mediate interactions with DNA or RNA, the RING motif is exclusively seen in protein–protein interactions and E3 ligases, enabling them to attach to E2 enzymes [3]. Based on the diverse combination of cysteine and histidine residue, the RING-zf domains were classified into 13 categories: RING-H2 (C3H2C3), RING-HC (C3HC4), RING-v, RING-D, RING-S/T, RING-G, RING-C2, RING-mH2, RING-mHC, C3HCHC2, C2HC5, C3GC3S and C2SHC [3, 4]. The RING-HC (C3HC4) can be further classified into two subtypes (RING-HCa and -HCb) [1]. RING-H2 and -HC have been found in practically every plant species, from algae to higher plants. Thus, they were considered as the two canonical RING-zf proteins that exhibited conserved functions and share conserved evolutionary process. For example, there were 241 RING-H2, 145 RING-HCa, and 41 RING-HCb in *Arabidopsis thaliana*, 281 RING-H2 and 119 RING-HC in rice, 367 RING-H2, 202 RING-HCa, and 6 RING-HCb in apple, 371 RING-H2, 215 RING-HCa, and 47 RING-HCb in *Brassica rapa*, 248 RING-H2, 142 RING-HCa, and 21 RING-HCb in *Solanum lycopersicum* (tomato), and 25 RING-H2, 26 RING-HCa, and 2 RING-HCb in *Ostreococus tauri* [3–5]. Interestingly, the other 11 RING-zf types are not fully included in every species except the two canonical types RING-H2 and -HC. The presence of RING-v, -C2, -S/T, and -G was only found in *A. thaliana*, apple, *B. rapa*, and tomato [3, 4]. RING-mH2 and -mHC were only identified in apple [4], C3HCHC2, C2HC5, C3GC3S and C2SHC4 were only identified in *O. tauri* [6]. In rice, as a *Poaceae*, RING-zfs consist of 281 RING-H2, 119 RING-HC, 23 RING-v, and 2 RING-C2 [4]. The classification of RING-zfs is highly variable, indicating that RING-zf has evolved from an independent way for different species.

Besides, more and more studies reveal that the canonical or non-canonical RING-containing proteins are E3 ubiquitin ligases, which function in their ubiquitinated target proteins to regulate diverse cellular activities, including auxin signaling, defense signaling, multiple abiotic stress response, etc. [2–4]. For example, in *A. thaliana*, COP1 (RING-HC) works as an E3 ubiquitin ligase directly binding to and degrading the transcription factor HY5 via the proteasome pathway in dark [7]. HOS1 (RING-C2) encodes an E3 ubiquitin ligase to mediate the ubiquitination of ICE1 (an ERF TF) for attenuating the cold stress response [8]. In rice, OsHIRP1 (RING-HC), as an E3 ubiquitin ligase, primarily targets to OsARK4 in the nucleus and OsHRK1 in the cytoplasm to increase heat resistance for plants [9]. The RING-HC (COP1, EMR, HUB1, AIRP1, and AIRP4) and RING-H2 (CIP8, BIG BROTHER, BRH1, FLY1, RFI2) in *A. thaliana*, RING-HC (OsHAF1, OsXB3.1 and OsRHC1) in rice, and RING-HC (LjCZF1) in *L. japonicas,* have been demonstrated to be important during plant development and disease resistance as E3 ubiquitin ligase responses [7, 10]. In tomato, 3 RING-vs (*Solyc07g41190*, *Solyc03g116030*, *Solyc04g054330*), 2 RING-C2s (*Solyc03g113700*, *Solyc03g033630*), and 1 RING-S/T (*Solyc02g91720*) strongly responded to salt stress and were significantly expressed in the ‘orange’ stage of fruit development [3]. In *T. aestivum*, several RING-zfs have been functionally validated. TaRZF70-RING-H2 has four distinct 4 RING-H2 ZF domains that are different from the other RING-zfs and show different responses to water shortage, i.e. up-regulation in leaves and down-regulation in roots [11]. The plants overexpressing *TaDIS1-RING-HC* had shorter roots compared with the wild type, and they were responsive to mannitol and ABA treatments [12]. The wheat expressing TaZnF-RING-HC showed enhanced resistance to dehydration and salt stress [13]. More TaRING-zfs need to be characterized to facilitate the creation of higher quality *T. aestivum*.

More than one-third of the world's population relies on *T. aestivum* as a staple diet [13]. Due to the hexaploid genome and substantial gene redundancy, the functions of *TaRING-zf* genes in *T. aestivum* compared to other crops such as rice and tomato are poorly understood. In the present study, we identified a set of TaRING-zfs families in the *T. aestivum* genome, dissected their classification, speculated their physicochemical properties and gene expression patterns. These results could provide more useful information on the evolution and functional elucidation of *RING-zf* genes in *T. aestivum*.

## Results

### Genome-wide identification of TaRING-zf genes

Based on the data about genome sequence and protein domain from the pfam (PF14634, E-value < 1e-05) and the Ensembl plants database, 138 putative RING-zfs were finally identified. The 138 *TaRING-zfs* varied from 123 (*TraesCS6B02G164200.1*) to 1041 (*TraesCS2B02G504600.1*) amino acids (aa) in length. The comprehensive analyses of TaRING-zfs including gene IDs, protein length, physical position, molecular weight, theoretical pI, atomic composition, instability indexes, aliphatic indexes, GRAVY values, and subcellular localization, were given in the Table S1.

**Fig. 1:**
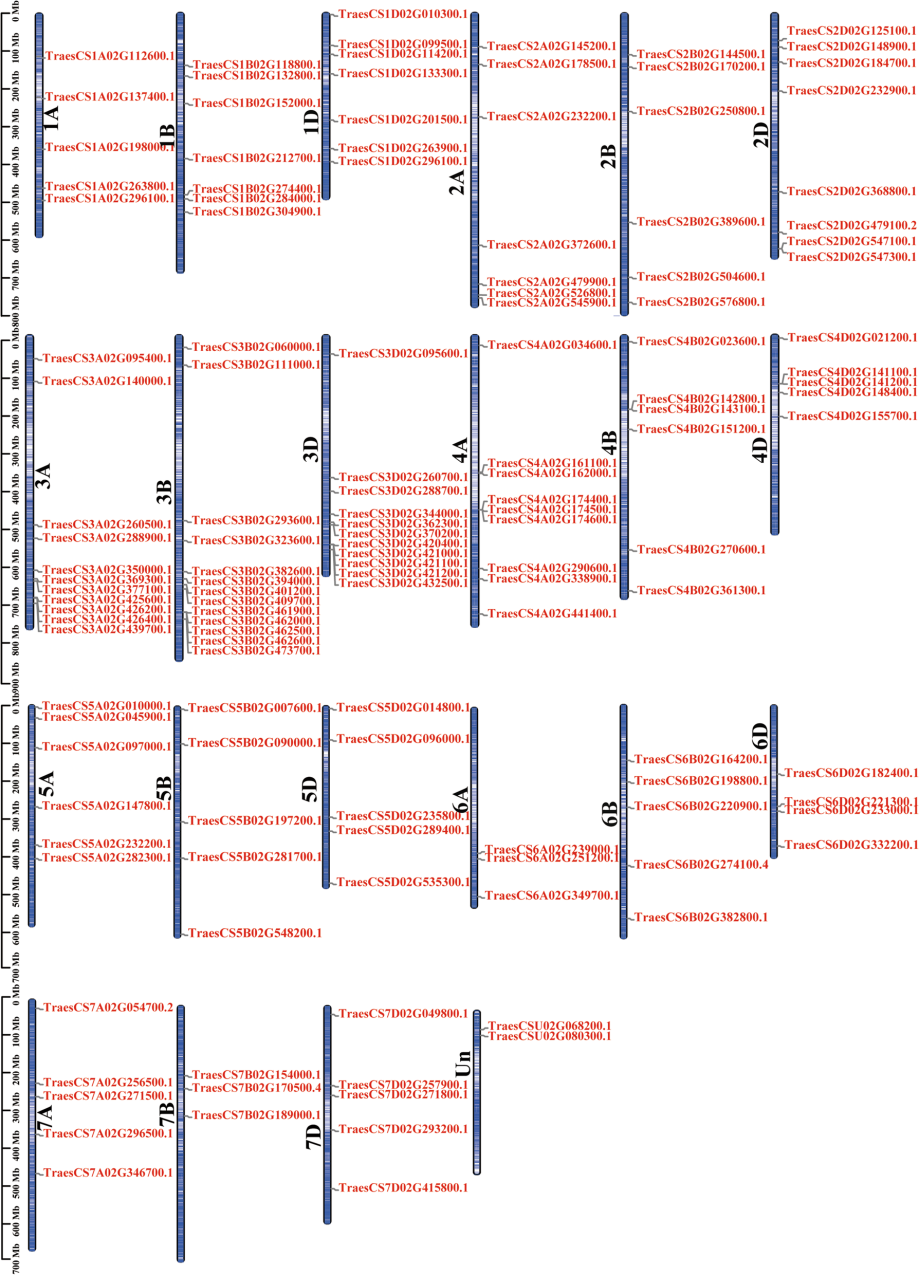
Chromosomal locations of 138 *TaRING-zf* genes in *T. aestivum.* The ruler on the left represents the chromosome length. Chr: Chromosome. The information of the starting and ending location for the 138 *TaRING-zf* genes are listed in the Table S1

According to the genome annotation file (GFF3), the chromosomal distribution of the *TaRING-zf* members of *T. aestivum* was analyzed. 138 *TaRING-zf* genes were averagely distributed on 7*3 (A, B, D) + Un chromosomes (chr) (Fig. 1). The *T. aestivum* 1 to 7, and Un chromosomes groups had 19, 21, 35, 20, 16, 12, 13, and 2 *TaRING-zf* genes, respectively (Fig. 1).

**Fig. 2:**
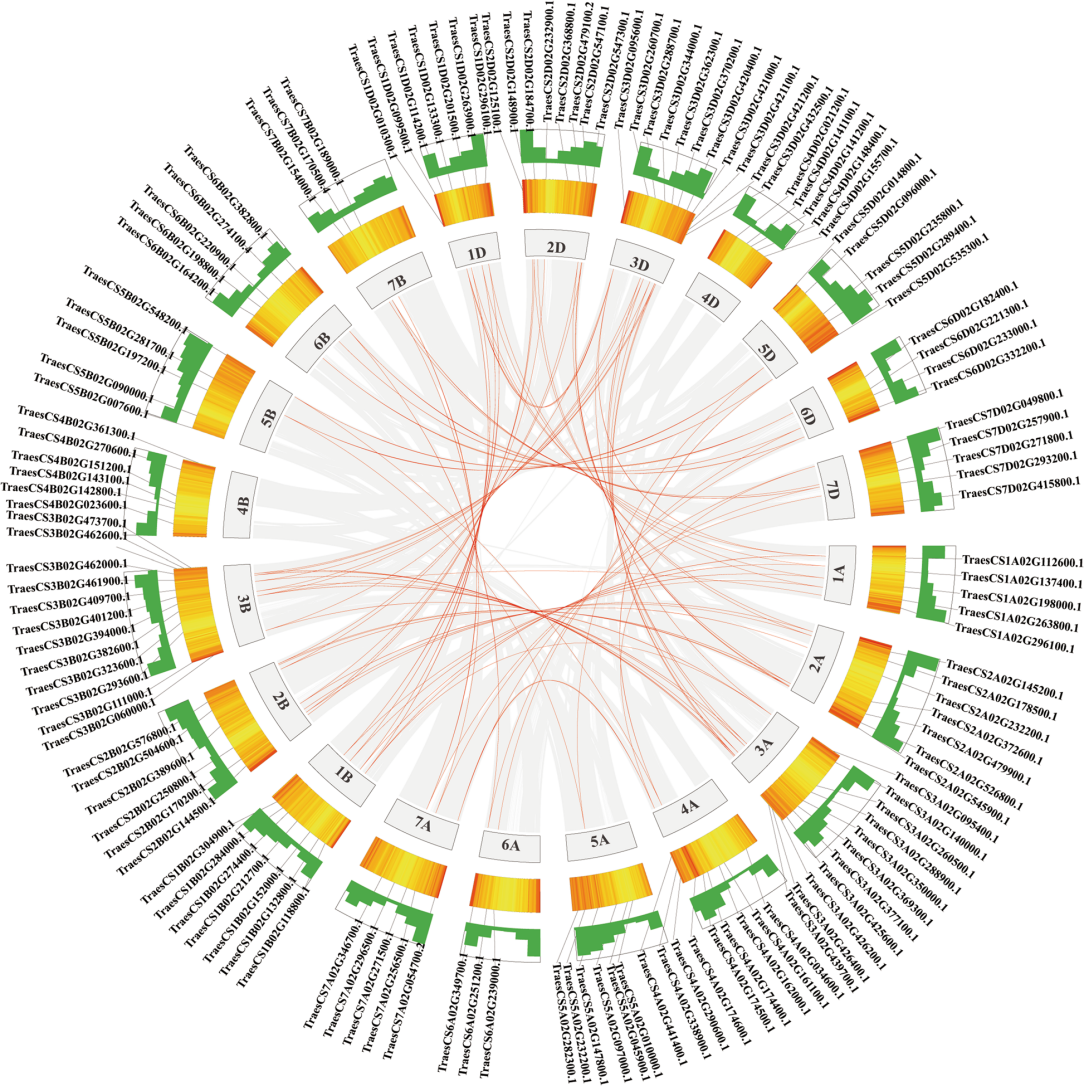
Schematic representations of the interchromosomal relationships of *T. aestivum. RING-zf* genes Colored lines indicate all synteny blocks in the *T. aestivum* genome, and the red lines indicate the duplicated *TaRING-zf* gene pairs. The chromosome number is indicated below each chromosome

A collinearity analysis was undertaken to further examine the duplication events in *TaRING-zf* genes. In 138 *TaRING-zf* genes, 82 pairs of fragment duplication genes were discovered (Fig. 2). TaRING-zf fragment duplication genes were predominantly found on chr 1 (16), chr 2 (18), and chr 3 (37) (A, B, and D), followed by chr 4A (4), chr 5 (4), chr 6 (6), and chr 7 (7) (A, B, and D), with no fragment duplication gene pairs on chr 4 (B and D) (Fig. 2).

According to subcellular localization prediction, 129 (93.5%) TaRING-zfs were located in nucleus, while only 4 (2.9%) proteins were located in both chloroplast and nucleus, and 3 (2.2%) were located in both cell membrane and nucleus. Only 1 (0.7%) (*TraesCS2A02G526800.1*) were located in chloroplast and 1 (0.7%) (*TraesCS2A02G178500.1*) were located in Golgi apparatus regions (Table 1). TaRING-zf proteins are most likely involved in the degradation of transcription factors or other nuclear expression proteins since they were mostly found in the nucleus.

**Table 1:** The subcellular localization result of 138 RING-zfs in *T. aestivum*

Postion of subcellular localization	Number of genes	Percentage of genes
Cell nucleus	129	93.5%
Chloroplast	1	0.7%
Golgi apparatus	1	0.7%
Chloroplast and nucleus	4	2.9%
Cell membrane and nucleus	3	2.2%

### A novel modified subtype RING-M was identified in *T. aestivum*

**Fig. 3:**
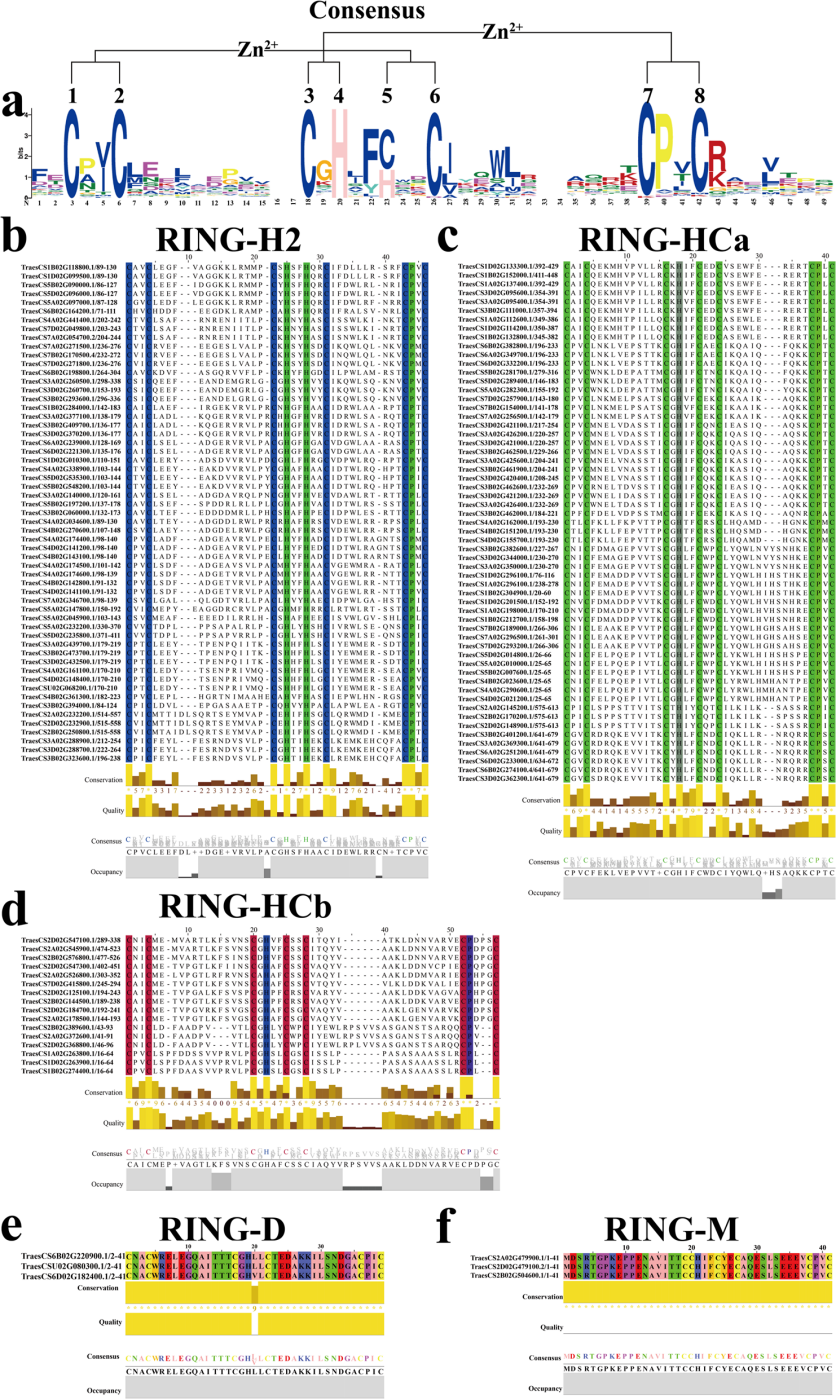
The conserved motif logo and classify of TaRING-zf family. **a** Sequence logos for the TaRING-zf motifs of *T. aestivum*. Numbers on the x-axis represent the sequence conservation of that position. The y-axis represents the relative frequency of the TaRING-zf motif amino acid of that position. The sequence logos was generated using MEME server. **b** The conserved functional sequences and genes of TaRING-H2 subsets proteins were predicted by MEME software. **c** The conserved functional sequences and genes of TaRING-HCa subsets proteins were predicted by MEME software. **d** The conserved functional sequences and genes of TaRING-HCb subsets proteins were predicted by MEME software. **e** The conserved functional sequences and genes of TaRING-D subsets proteins were predicted by MEME software. **f** The conserved functional sequences and genes of TaRING-M subsets proteins were predicted by MEME software

One hundred thirty-eight RING-zf domains were separated into four RING categories according to the type of the metal ligand residues and/or the quantity of amino acids, including 57 RING-H2 and 75 RING-HC (59 RING-HCa and 16 RING-HCb), 3 RING-D and 3 novel RING-M (Fig. 3). The alignment of full length of 138 TaRING-zf proteins can be found in Figure S1. Interestingly, such RING-zf domains, including RING-(v, C2, S/T, G, mH2, and mHC), C3HCHC2, C2HC5, C3GC3S, and C2SHC, were not detected in the *T. aestivum* [1, 3–5, 14].

Importantly, we identified a novel RING-M domain in *T. aestivum*, which was not reported in the previous studies. The consensus sequences of RING-M domain was described as M-X_2_-R-X_14_-Cys-X_1_-H-X_2_-Cys-X_2_-Cys–X_10_-Cys–X_2_-Cys. The alterations in the RING-M type domain were located in the first zinc active center, with Met (M) and Arg (R) at the metal ligand positions 1 and 2, respectively. The RING-M domain featured a revised layout with conventional RING-HC type metal ligands at positions 3 and 4 (Fig. 3f). These results cast a new light on RING-zf classification.

Moreover, the canonical RING-HC domain in *T. aestivum*, 59 (78.7%) of 75 the TaRING-HC were RING-HCa type, while only 16 belonged to RING-HCb (Fig. 3c, d). The proportion of RING-HCa is significantly higher than RING-HCb, consistent with other species [1, 3–5, 14]. Furthermore, the conserved spacing way in diverse RING-zf domain was basically same with other species (Fig. 3b, d, e) [3].

### Phylogenetic, motif and structural analysis of TaRING-zfs

**Fig. 4:**
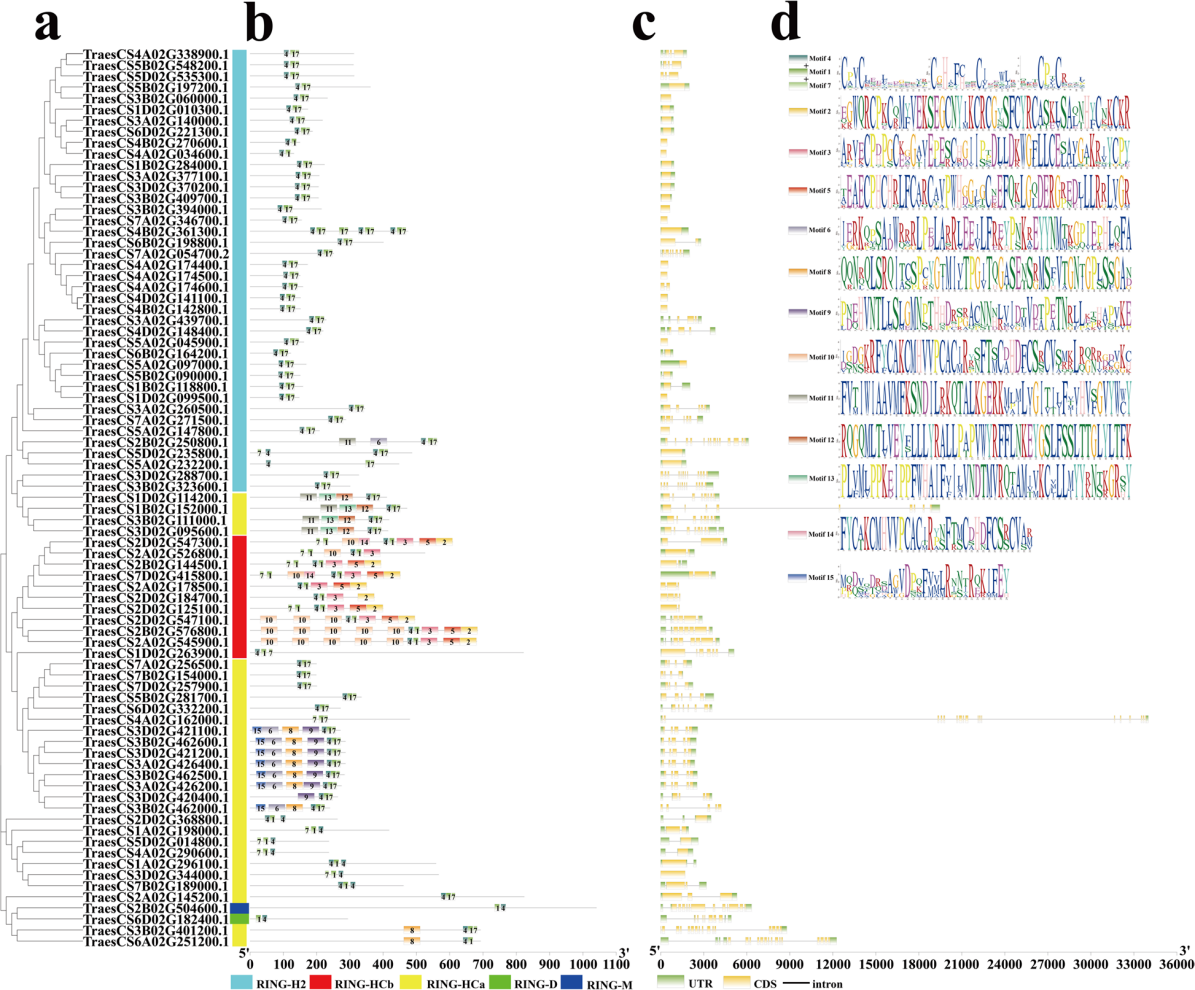
Phylogenetic relationships, gene structure and architecture of the conserved motifs of 80 TaRING-zfs in *T. aestivum*. **a** The phylogenetic tree was constructed based on the full-length sequences of TaRING-zf proteins using the MEGA7.0 software. **b** The motif composition of TaRING-zf proteins. The motifs, numbers 1–15, are displayed in different colored boxes. **c** Exon, intron, and UTR structure of 80 *TaRING-zf* genes. Yellow boxes indicate untranslated 5’ and 3’ regions, green boxes indicate exon, and black lines indicate introns. The gene full length and protein length can be estimated by using the scale at the bottom. **d** Schematic display of 15 conserved motifs in 80 TaRING-zf proteins

The 80 non-redundant domains were retrieved using the CD-HIT program (cutoff > 95%) (Fig. 4a). By further comparing the genomic DNA sequences of TaRING-zfs, the exon, intron, UTR structure, as well as motif characteristics of TaRING-zfs were annotated for better understanding the structural composition of TaRING-zfs. However, there is no clear distinction between the different subtypes in terms of the characteristics of the genome structure (Fig. 4b, c and Fig. S2). An unrooted phylogenetic tree of 80 TaRING-zf conserved domains was constructed. The majority of RING-HC, -H2, -D, and -M were well separated (Fig. 4a). However, RING-D (TraesCS6D02G182400.1) domain and RING-M (TraesCS2B02G504600.1) were grouped with RING-HC group.

The coding sequences of most TaRING-zf genes contain 1 to 19 exons (Fig. 4c). Several *TaRING-zf* genes contain very long introns, e.g. *TraesCS4A02G162000.1* and *TraesCS1B02G152000.1* with up to 32.3 kb and 19.6 kb introns, respectively (Fig. 4c).

The motifs of full-length TaRING-zfs were analyzed by the MEME software (Fig. 4b, d and Fig. S2c). The schematic diagram was created for describing the structure of TaRING-zf proteins based on the results of the motif analysis. We identified a total of 15 conserved motifs in them (Fig. 4b, d). The motif-4, 1 and 7 were constituted the RING-H2 and RING-HCa domains, motif-4,1 and 3 were constituted the RING-HCb domain, and motif-4 and 1 were constituted the RING-D and RING-M domains (Fig. 4b, d).

Other than the RING-zf domain, 10 different types of protein domains were discovered in *T. aestivum* RING-zf proteins (Fig. S3). These predicted additional conserved domains may be considered to be possible PPI domains involving in substrate recognition, such as a coiled coil domain and WD-40 (Fig. S3c, g). DEXDc and HIRAN were identified as the motifs with nucleic acid binding ability, which linked to the RING domain (Fig. S3f). ZnF-CHY and Zinc ribbon 6 domains were expected to work in metal-ion binding (Fig. S3e). The IBR domain (Fig. S3b) [15] is a C6HC-type zinc finger motif found in 17 RING-HCb. The consensus sequence for the IBR domain is C-X_(4)_-C-X_(14–30)_-C-X_(1–4)_ -C-X_(4)_ -C-X_(2)_-C-X_(4)_-H–X_(4)_-C and is one of the four zinc-binding RING, LIM, and PHD finger family members [16]. In *A. thaliana*, the IBR domain is exclusive to the ARI family [16]. One or more transmembrane (TM) segments are found in 29 TaRING-zf proteins (Fig. S3a). To far, the two domains related with the RING-zf motif have only been discovered in *T. aestivum*, including RPT1, pfam Lon-substr-bdg and HELICc domains (Fig. S3d). The motif analysis revealed that 19 TaRING-zf proteins contained 2 to 5 RING-zf domains (Fig. S3a, b, c, h). Previous research demonstrated that the majority of new domains were also discovered in other plant species, such as tomato, *B. rapa*, *O. tauri*, apple, *A. thaliana*, and so on, implying that the role of these additional domains may be conserved among them [1, 3–5, 16].

### *T. aestivum's* RING-zf gene family had a closer evolutionary link with Poaceae

**Fig. 5:**
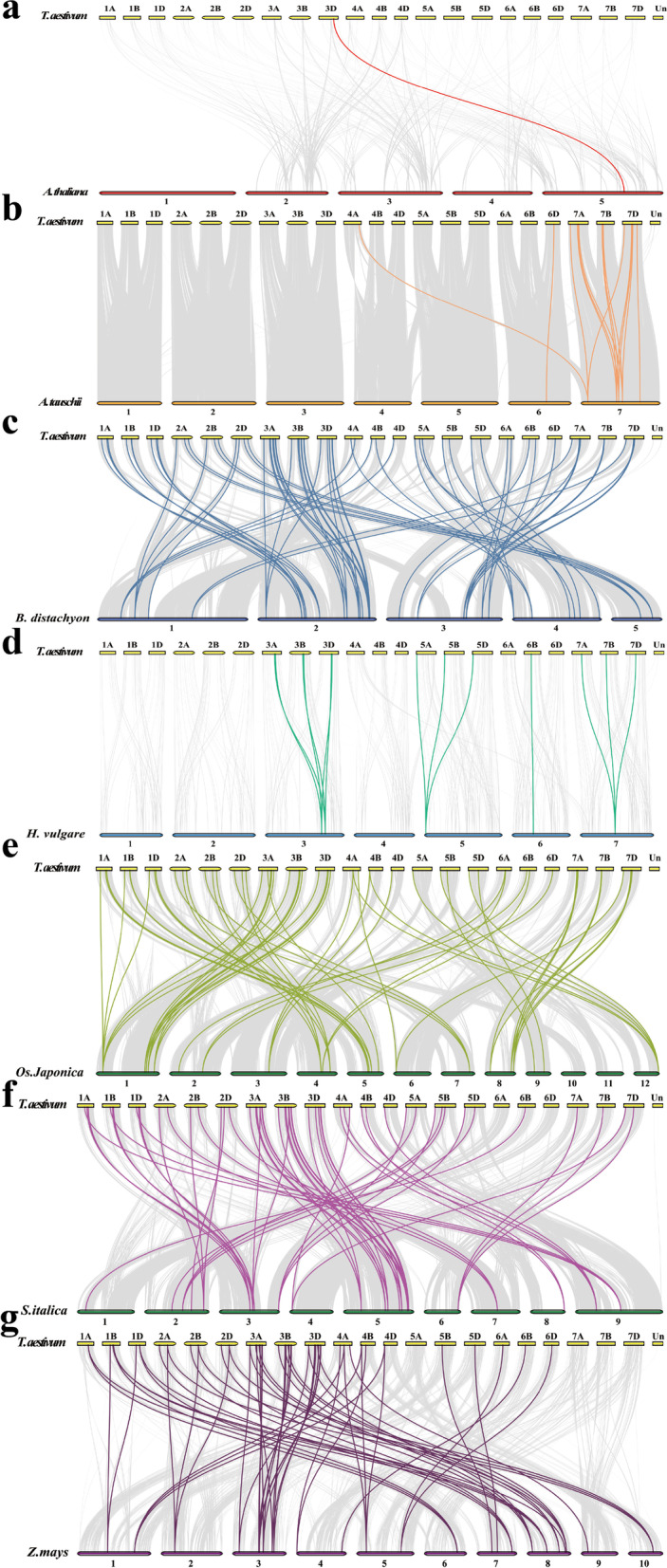
Collinearity analysis of *TaRING-zf* genes. **a** Colinearity analysis of *TaRING-zf* genes with *Arabidopsis thaliana*, (**b**) Colinearity analysis of *TaRING-zf* genes with *Aegilops tauschii*, (**c**) Colinearity analysis of *TaRING-zf* genes with *Brachypodium distachyon*, (**d**) Colinearity analysis of *TaRING-zf* genes with *Hordeum vulgare*, (**e**) Colinearity analysis of *TaRING-zf* genes with *Oryza sativa Japonica*-rice, (**f**) Colinearity analysis of *TaRING-zf* genes with *Setaria italic*, (**g**) Colinearity analysis of *TaRING-zf* genes with *Zea mays*. The light background represents synteny blocks in the whole genome of the seven species, and dark lines represent collinear gene pairs of TaRING-zf

The effective information about the evolutionary relationship among species can be provided by orthologous gene pairs [17]. The *TaRING*-*zf* genes with a close relationship in different plants shared similar domain composition. Therefor, the syntenic maps of *T. aestivum* related with other 6 monocots (*A. tauschii*, *B. distachyon*, *H. vulgare*, *O. Sativa*, *S. italica*, and *Z. mays*) as well as 1 dicot (*A. thaliana*) were further constructed (Fig. 5). We detected 89, 66, 13, 57, 55, 56, and 1 gene pairs in *A. tauschii*, *B. distachyon*, *H. vulgare*, *O Sativa*, *S. italica*, *Z. mays*, and *A. thaliana*, respectively. Some *TaRING-zf* genes, such as *TraesCS1A02G296100* and *TraesCS1B02G304900*, were discovered to be related with at least two pairs of homologous genes (particularly between wheat and maize), suggesting the essential roles of these genes in the TaRING-zf family during evolution (Table S2). Furthermore, their findings demonstrated that the *TaRING-zf* gene family was highly conserved, with *TaRING-zf* genes being closer to monocot genes than dicot genes (Fig. 5). A common ancestor gene could be shared by the *TaRING-zf* genes in several plants.

The Ka/Ks average ratios among *T. aestivum* and the *6* monocots were calculated to be 0.39, 0.30, 0.29, 0.31, 0.28, and 0.26 (Table S2). All of the collinear gene pairings had a value less than one, suggesting that the TaRING-zf family evolved under strong purifying selection in *T. aestivum*. The divergence time of collinear gene pairs in *S.italica* was approximately 50 Mya, consistent with that of *Z. mays*, and earlier than that in *A. tauschii* (14.61 Mya), *B. distachyon* (38.92 Mya), and *H. vulgare* (46.54 Mya), indicating that the TaRING-zf family was closely related to those in *A. tauschii*, *B. distachyon*, *H. vulgare*, *O. Sativa*, *S. italica* as well as *Z. mays*.

### Cis-element analysis of TaRING-zf gene promoters

**Fig. 6:**
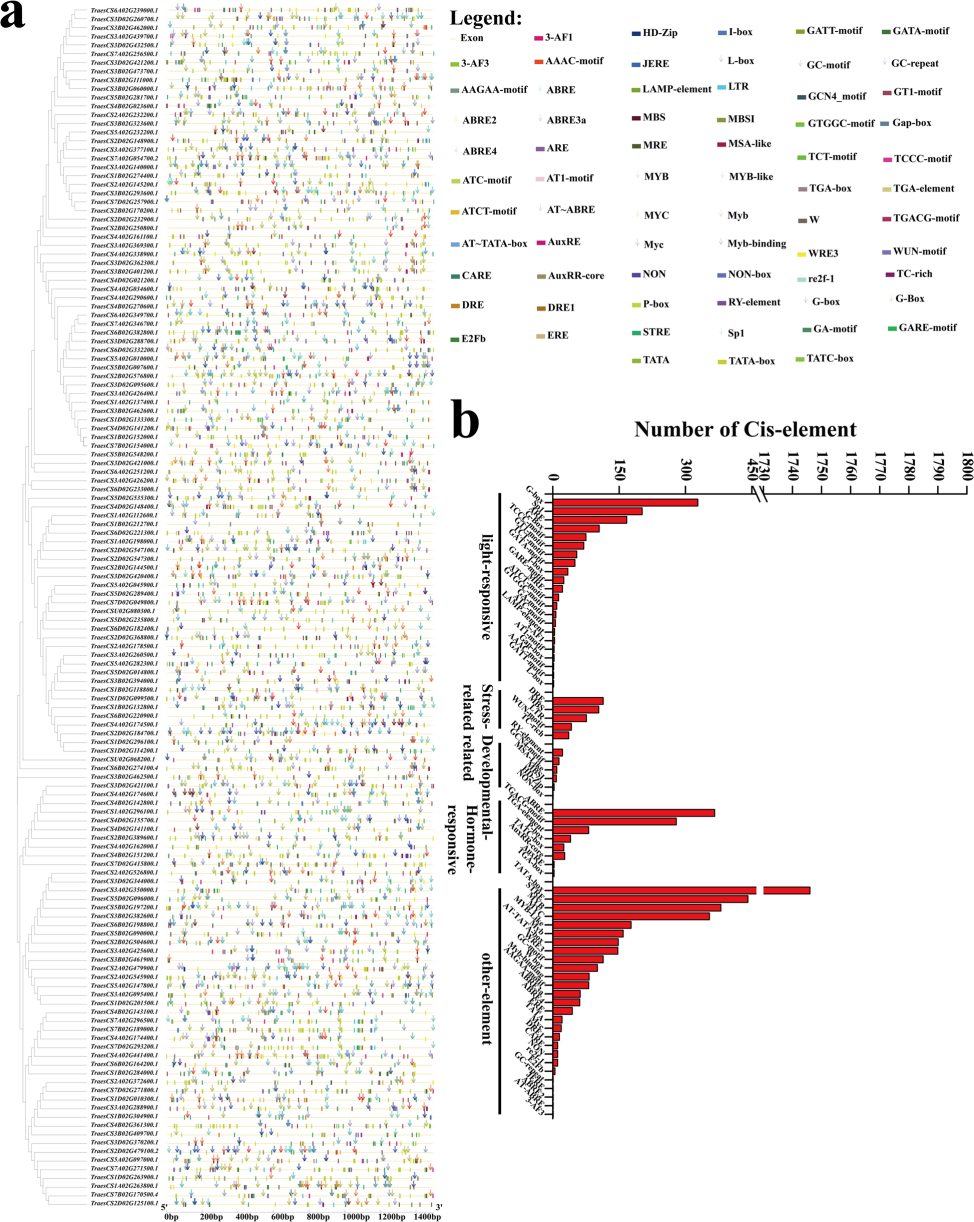
The information of *cis*-acting elements on the promoters of *T. aestivum RING-zf* genes. **a** The *cis*-acting elements on the promoters of *T. aestivum RING-zf* genes. A variety of types *cis*-elements–transcription-related, development-related, hormone-related, and abiotic stress-related elements were identified in the *TaRING-zf* gene promoter regions. All *cis*-elements in the *TaRING-zf* gene promoter are listed in Table S2. **b** Types and number of *cis*-acting regulatory elements analysis involved in the growth, development, stress and hormonal response

Further investigation of the cis-elements in the *TaRING-zf* gene promoters was important for understanding the regulatory functions of genes. The 1500 bp flagment upstream of the 5' end of 138 *TaRING-zf* genes were analysized using the in Plant CARE. There were 68 types (6635) of *cis*-elements in 138 *TaRING-zf* gene promoter regions, which included elements related to light response, stress response, developmental and hormonal response, and other unknown factors (Fig. 6a, b, Table S3).

The five types of stress-related elements were identified in *TaRING-zf* genes, including 82 (59.4%) DRE, 65 (47.1%) MBS, 59 (42.8%) LTR, 35 (25.4%) WUN-motif and 33 (23.9%) TC-rich elements (Fig. 6b and Table S3). Among six kinds of development-related elements, RY-element (12) was the largest number of regulatory elements involved in seed-specific regulation (Fig. 6a and Table S3). There are 8 types of hormone related elements, of which 120 (86.9%) have *cis*-elements implicated in ABA response (ABRE-element), followed by TGA-elements involved in auxin response (59) (Fig. 6b and Table S3). Furthermore, five different types of environmental stress-related elements were discovered in 138 TaRING-zf gene promoter regions. It was worth noting that 137 *TaRING-zf* gene promoter regions contained 1–15 types, suggesting that TaRING-zfs may be related to the responses to light stresses (Fig. 6a and Table S3). The G-box, ARE, Sp1 and G-Box existed in 112 (81.2%), 83 (60.1%), 83 (60.1%), 83 (60.1%) and 66 (47.8%) *TaRING-zf* genes, respectively (Fig. 6b and Table S3). The details of the number and type of other *cis*-elements was showed in Table S3. Distinct types and amounts of response cis-elements were found in varied *TaRING-zf* gene promoter regions, suggesting that TaRING-zfs were engaged in growth and development and had different regulation mechanisms in response to diverse stress and hormone treatments.

### Expression profiles of TaRING-zf genes

**Fig. 7:**
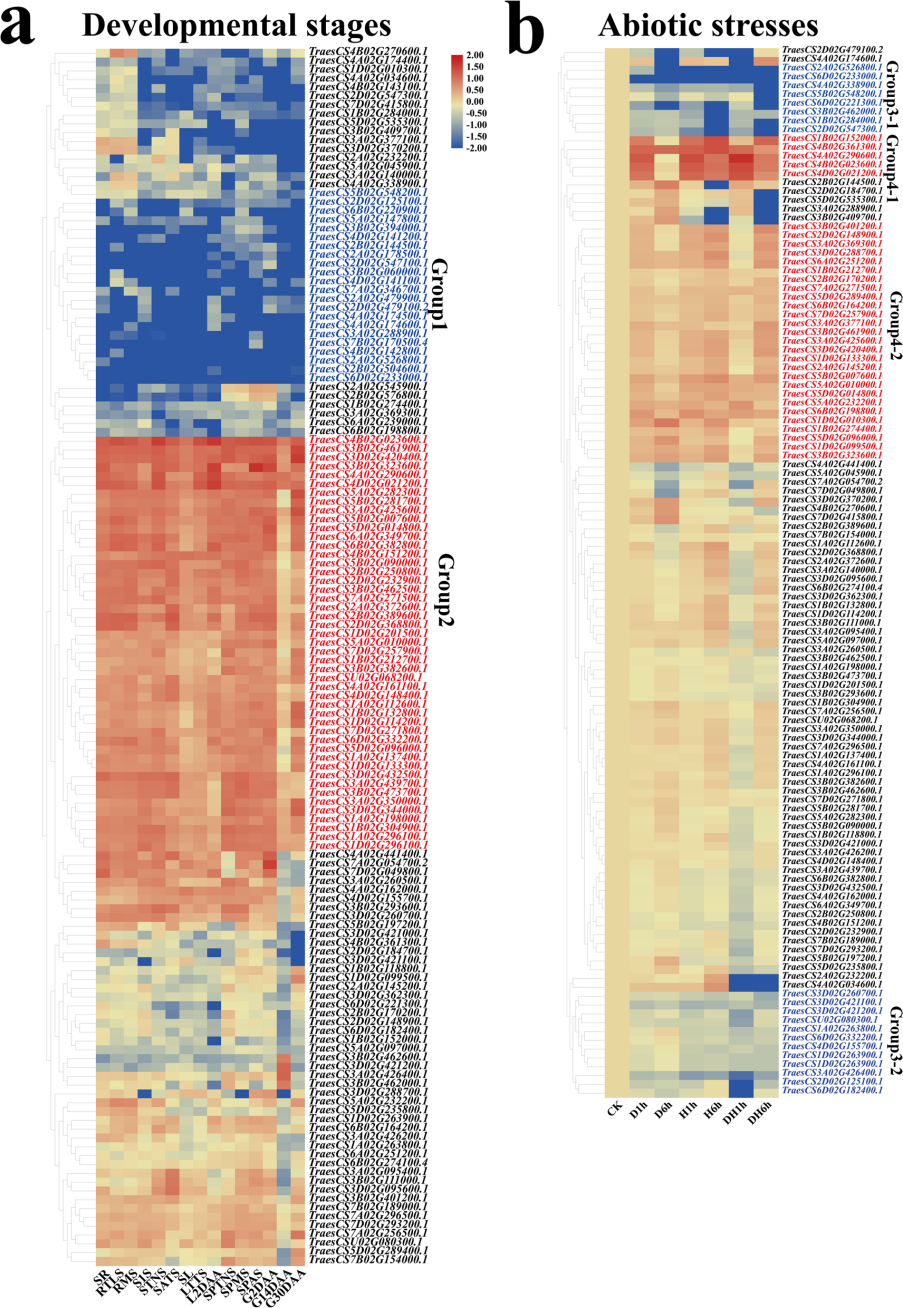
Transcriptome analysis of *TaRING-zf* genes. **a** Expression profiles of 138 *TaRING-zf* genes in different developmental stages. The different tissue types are shown on the bottom side: seedling roots (SR), roots at the three leaves (RTLS), roots at the meiosis (RMS), stems at the 1 cm spike (S1S), stems at the two nodes stems (STNS), stems at the anthesis stems (SATS), seedling leafs (SL), leafs at the three tillers (LTTS), leafs at the 2 days after anthesis (L2DAAs), spikes at the two nodes stems (SPTNS), spikes at the meiosis (SPMS), spikes at the anthesis stems (SPAS), grains at the 2 days after anthesis (G2DAAs), grains at the 14 days after anthesis (G14DAAs), grains at the 30 days after anthesis (G30DAAs). **b** Expression profiles of 119 *TaC3HC4-RING finger* genes under different abiotic stress treatments. The different treatment types are shown on the bottom side: drought 1 h (D1h), drought 6 h (D6h), heat 1 h (H1h), heat 6 h (H6h), drought and heat 1 h (DH1h), drought and heat 6 h (DH6h). Hierarchical clustering of the relative transcript abundance profiles of genes, and the warmer colors indicate the higher expression. The scale bar indicates relative expression level as shown in the middle of the two heat maps. The individual gene names are indicated on the right side

We further dissected the transcription level of *TaRING-zf* genes at 15 distinct developmental stages and under various abiotic stimuli such as drought, heat, and drought and heat using the Wheat Expression Browser (expVIP) (www.wheat-expression.com) (Fig. 7a, b and Table S4). The 22 (15.9%) and 47 (34.1%) *TaRING-zf* genes were constitutively expressed with high or low abundance in nearly all developmental stages, respectively (Fig. 7a, genes labeled in Group 1 and Group 2). The 20 (14.5%) and 27 (19.7%) *TaRING-zf* genes were transcripted with high or low abundance under heat, drought, and heat and drought stress treatment, respectively (Fig. 7b, genes labeled in Group 3 and Group 4). The results suggested that some *TaRING-zf* genes play potential functions during wheat development and response to heat, drought, and heat and drought stress.

**Fig. 8:**
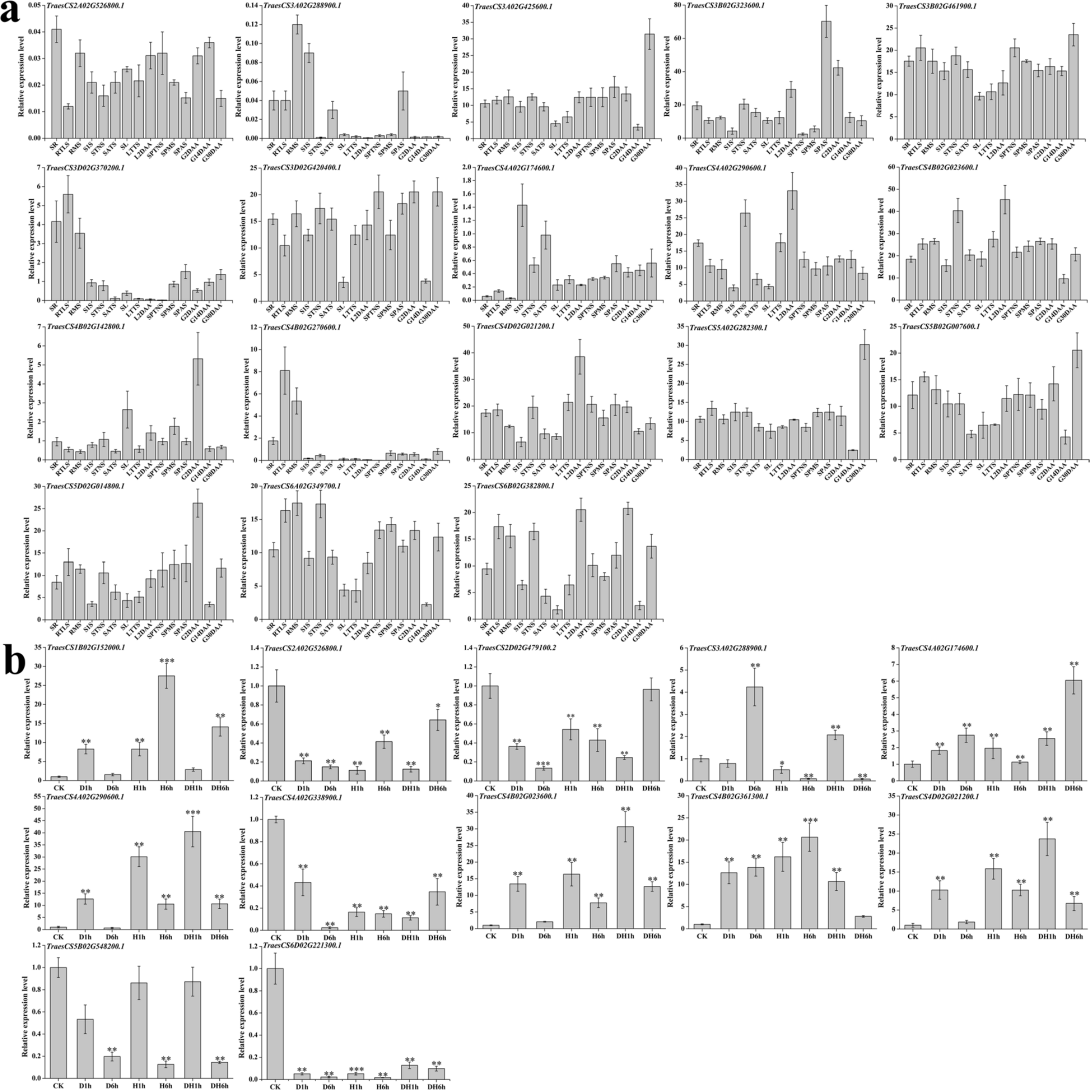
Quantitative RT-PCR analysis of 30 *TaRING-zf* genes. **a** Relative expression levels of 18 *TaRING-zf* genes in 15 developmetal stages (seedling roots (SR), roots at the three leaves (RTLS), roots at the meiosis (RMS), stems at the 1 cm spike (S1S), stems at the two nodes stems (STNS), stems at the anthesis stems (SATS), seedling leafs (SL), leafs at the three tillers (LTTS), leafs at the 2 days after anthesis (L2DAAs), spikes at the two nodes stems (SPTNS), spikes at the meiosis (SPMS), spikes at the anthesis stems (SPAS), grains at the 2 days after anthesis (G2DAAs), grains at the 14 days after anthesis (G14DAAs), grains at the 30 days after anthesis (G30DAAs)). **b** relative expression levels of 12 *TaRING-zf* genes under 3 different stress treatments (Check (CK), drought 1 h (D1h), drought 6 h (D6h), heat 1 h (H1h), heat 6 h (H6h), drought and heat 1 h (DH1h), drought and heat 6 h (DH6h)). Vertical bars indicate standard deviation

Based on these findings, 30 representative genes (18 from different developmental stages and 12 from abiotic stress) were demonstrated using qPCR (Fig. 8a, b). For example, TraesCS3D02G370200.1 expression levels, were down-regulated throughout the stem and leaf growth stages, but rose during grain development. The transcription of *TraesCS5D02G014800.1* was up-regulated at the development stages of root, leaf and spike. *TraesCS4A02G290600.1* and *TraesCS3B02G323600.1* was activited transcriptionally during the development leaf and spike, but reduced in root and grain (Fig. 8a, b). Overall, the TaRING-zf family may have critical roles in different tissues and developmental stages of *T. aestivum*.

The qPCR was used to analyze the expression patterns of the 12 *TaRING-zf* genes during drought and heat stress. Overall, some *TaRING-zf* genes were highly induced/repressed by different stress treatments. For instance, *TraesCS1B02G152000.1*, *2A02G526800.1*, *4A02G290600.1*, *4A02G338900.1*, *4B02G361300.1*, and *6D02G221300.1* significantly responded to all stress treatments. Interestingly, drought and heat stress treatments reduced the transcript levels of numerous *TaRING-zf* genes, including *TraesCS2A02G526800.1*, *2D02G479100.2*, *4A02G338900.1*, and *6D02G221300.1*. However, various treatments resulted in conflicting expression patterns for numerous genes. For example, *TraesCS3A02G288900.1*, was strongly increased by D6h and DH1h treatments, but repressed by H1h and H6h treatments (Fig. 8b).

## Discussion

### A novel RING-M subtype was identified in *T. aestivum*

RING-zf genes are widespread in a diverse range of plant species [3]. However, there are few studies on the RING-zf family in *T. aestivum*. The genome-wide investigation identified 138 RING-zf family members in *T. aestivum* genome, which was essential to better understand the function of the *RING-zf* genes in wheat.

The TaRING-zf domains were divided into four different sub-types (RING-H2, -HC (RING-HCa/b), -D as well as -M). The proportion of RING-HCa was significantly greater than RING-HCb, which was consistent with other species, such as 186 RING-HCs in *A. thaliana*, with 145 (77.9%) proteins belonged to RING-HCa. In *B. rapa*, 215 (80.1%) of 262 RING-HCs belonged to RING-HCa. In tomato, 142 (87.1%) of 163 RING-HCs belonged to RING-HCa. In *O. tauri*, 26 (92.9%) of 28 RING-HCs belonged to RING-HCa. In apple, 202 (97.1%) of 208 RING-HCs belonged to RING-HCa [1, 3–5, 14]. These results suggest that the RING-HC subtype may play dominant roles.

The spacing between the eight zinc-coordinating residues and their properties RING-D types were also observed in the described apple and *B. rapa*, which were not *A. thaliana* -specific [4, 5]. However, this novel modified RING-M domain was demonstrated to be specific in *T. aestivum*, which was not detected in other genomes. The alterations in the RING-M type domain occur in the first zinc active center, with Met and Arg at the metal ligand positions 1 and 2, respectively. The RING-M domain featured a revised layout with conventional RING-HC type metal ligands at positions 3 and 4 (Fig. 3f). RING-M (TraesCS2B02G504600.1) gene was showed the syntenic relationship with those in *B. distachyon* (RING-HC, KQJ84776), rice (RING-HC, Os04t0629300-02) (Fig. 5 and Table S3). It can also be observed from the phylogenetic tree that RING-M may have evolved from RING-HC (Fig. 4a). The unique RING-C2HC5 domain in *O. tauri* has a revised arrangement at metal ligand positions 3 and 4 also with classical RING-HC type, as well these RING-mH2 and -mHC domains in apple have the substitutions happening in the 2nd zinc coordinating center with Gly (G), F (phe), Y (Tyr), L (Leu), P (Pro) and Q (Gln) at the metal ligand positions 8. The E3 activity of the RING-M, -C2HC5, -mH2, and -mHC domains has yet to be proved experimentally, while the conserved metal ligand residues outside the changed position, as well as the highly conserved spacing between each location, suggested that these protein groups may have E3 activity. Moreover, RING-v and -C2 were detected in *O. Sativa* [4], but no RING-v and -C2 were identified in *T. aestivum*. These results indicate that RING-v and -C2 may occurred after the evolution of the *Poaceae*.

### Syntenic analysis demonstrated that TaRING-zf gene family were more closely evolutionary relationship with Poaceae

Intron/exon structural variations are known to be created by insertion/deletion events, which helps to evaluate the evolution trends of gene families [18]. Introns are thought to be under low selection pressure. In our study, all 138 *TaRING-zf* genes have introns (Fig. 4c), indicating that these genes have developed rapidly. Because of its hexaploid genome and high gene redundancy, *T. aestivum* genomes have a larger fraction of duplicated genes than other plants. The chief drivers of these expansions are tandem, segmental, and whole-genome duplication events [19]. The study discovered that in the *T. aestivum* genome, 59.4% of *TaRING-zf* genes were grouped as duplicated genes (Fig. 2). This indicates that segmental duplication, rather than tandem duplication, was responsible for the growth of the *T. aestivum* RING-zf family.

The evolutionary processes of the TaRING-zf family were explored further. *T. aestivum* comparative syntenic maps were created for seven representative species, including one dicot and six monocots (Fig. 6). We only detected 1 gene pair between *T. aestivum* and *A. tauschii*. It was detected more than 50 gene pairs between wheat and other plants, except for 13 gene pairs between *T.aestivum* and *H. vulgare*, suggesting that TaRING-zfs had a close relationship with *RING-zf* genes in *Poaceae* (Table S3). Two TaRING-zf collinear gene pairs (*TraesCS3A02G377100* and *TraesCS6B02G198800*) were only identified among *T. aestivum*, *A. tauschii*, *B. distachyon*, *H. vulgare*, *O. Sativa*, *S. italica* as well as *Z. mays,* which were not identified in *T. aestivum* and *A. thaliana*, such as *TraesCS3A02G377100*/*AET3Gv20841900*/*KQK10758* and *TraesCS6B02G198800*/*AET6Gv20432200*/*KQJ93775*. It may reveal that the these orthologous pairs were produced after the divergence of dicots and monocots plants (Table S3). Twelve TaRING-zf collinear gene pairs (eg: *TraesCS1A02G296100* and *TraesCS2A02G479900*) were only identified among *T. aestivum*, *A. tauschii*, *B. distachyon*, *O. Sativa*, *S. italica* as well as *Z. mays,* which were not found between *T. aestivum*, *A. thaliana* and *H. vulgare*. Furthermore, one syntenic pair was discovered between *T. aestivum*, *A.thaliana*, *B. distachyon*, *S. italica*, and *Z. mays* (*TraesCS3D02G420400*/*AT5G48655*/*KQK11225*/*KQL08330*/*Zm00001d042350T002*), showing that these orthologous pairs could existed before the ancestral divergence.

Next, using the Ka/Ks ratio to understand the direction and magnitude of natural selection activities on various protein-coding genes, recent studies reveal that the means of Ka and Ks for *T.aestivum* and other Poaceae were very close, and *TaRING-zf* genes underwent strong purifying selection [20]. It suggested that the *TaRING-zf* gene family's evolutionary process may be comparable to those of other plant gene families. The results are consistent with some WRKY collinear gene pairs in pineapple, and they were also formed before or after the divergence of dicots and monocots plants [21]. Compared with other genes family, TaRING-zf family is more stable and conserved, and it is essential for plants survival under serious various stress.

### Several TaRING-zf candidate genes may contribute to development and abiotic stress responses

Previous reports have revealed that the *RING-zf* genes play key roles during plant development as well as response to abiotic stresses [2, 10, 13, 21]. We meticulously investigated the expression profiles of *TaRING-zf* genes as well as their promoter elements of these genes. Combined with these results, we screened some proteins that may be involved in development and stress response. In this study, 6 candidate genes were identified in this investigation as potentially important in plant response to diverse stresses, including 4 RING-HC (*TraesCS2A02G526800.1*-*TaR2A5268*, *TraesCS4A02G290600.1*-*TaR4A2906 TraesCS4B02G023600.1*-*TaR4B0236* and *TraesCS4D02G021200.1*-*TaR4D0212*) and 2 RING-H2 (*TraesCS3A02G288900.1*-*TaR3A2889* and *TraesCS4A02G174600.1*-*TaR4A1746*) (Figs. 5, 7, 8 and Table S2). The *TaR4A2906*, *TaR4B0236*, *TaR4D0212*, *TaR3A2889* and *TaR4A1746* were response to drought stress by expression pattern analyze and qRT-RT verification (Figs. 7a and 8a), In addition, the MBS, DRE and ABRE elements were found in the promoters of five candidate genes (Fig. 5 and Table S2) [22–25]. It is widely understood that ABA-independent and ABA-dependent signaling pathways engage in the drought stress response and influence stress gene expression [26–28]. DRIP1 and DRIP2 are E3 ubiquitin ligases in *A. thaliana* that can regulate the ubiquitination of DREB2A. They negatively regulate the drought-responsive gene expression by directing DREB2A for proteolysis by the 26S proteasome [7, 29]. The *TaR4A2906*, *TaR4B0236*, and *TaR4D0212* were homologous to *DRIP1* and *DRIP2*, five *RING-zf* genes belong to the same subsets (RING-HC) and all of them were located in the nucleus. The promoter regions of the five *RING-zf* genes all contain the drought *cis*-regulatory element (ABRE) (Fig. 5a, Tables S1 and S2). *TaR3A2889* and *TaR4A1746* were homologous to rice OsDIS1, three *RING-zf* genes belongs to the RING-H2 subtype and was located in the nucleus. OsDIS1 negatively controls the drought stress tolerance through transcriptional regulation or possible post-translational regulation of rice OsNek6 [30]. The promoter regions of three *RING-zf* genes all contain the drought *cis*-regulatory element (DRE). Moreover, these five genes were significantly up-regulated at D1h or D6h by qRT-PCR verify (Fig. 8b), The result demontated that five genes may employ important role as E3 ubiquitin ligases in mediation of the transductions of drought signaling. Similarly, *TaR4A2906*, *TaR4B0236* and *TaR4D0212* were highly expressed in 5 development stages. The ABRE was detected in promoters of *TaR4A2906*, *TaR4B0236* and *TaR4D0212* (Fig. 5 and Table S2).

*TaDIS1* (RING-HC), a homologous gene of *TaR4A2906*, *TaR4B0236* and *TaR4D0212*, enhanced ABA hypersensitivity during seed germination and early growth and seedling development in *A. thaliana* [12]. The three genes were highly expression in grain stages by qRT-PCR verify (Fig. 8b). Notably, *TaR4B0236* was not only significantly expressed in different development stages, but also were significantly up-regulated drought stress. The promoter region of *TaR4B0236* contained both ABA (ABRE) and drought-responsive (MBS and DRE) *cis*-elements. These results demontated that *TaR4B0236* played a key important role in the signal pathway triggered by abiotic stress and developmental processes. Due to the large and complex genome of *T. aestivum*, we are poorly understood about the function of the *RING-zf* family genes. As a result, the preferentially expressed genes at distinct developmental and in response to drought and heat stress may merit special attention for further research into their physiological activities.

In this study, we conducted in-depth analyses through sequence alignment (key domains and amino acids), evolutionary relationship, expression pattern and qPCR verification. Compared with the RING-finger genes reported in other species with known functions, we identified six candidate genes that are likely to participate in plant development stages. 5 candidate genes were found to respond drought stress. At the same time, the promoters of 5 candidate genes were analyzed. The promoters of them contain the elements responsive to drought, dehydration, and ABA, indicating that they may participate in regulating drought tolerance of *T. aestivum* via these pathways. This study lays a solid foundation for further analyzing the molecular mechanism of wheat drought resistance, and provides new genetic resources for wheat drought resistance molecular design and breeding.

Overall, functional gene investigation in *T. aestivum* might aid with transgenic research to increase *T. aestivum* traits like the grain yield. The analysis of TaRING-zf structural domains and identification of *cis*-element will provide some key information for future dissection of the functions of *T. aestivum* RING-zf family members.

## Conclusion

A comprehensive analysis of TaRING-zf family in *T. aestivum* was performed in this study. The cellular localization of the 138 TaRING-zf proteins revealed that 129 (93.5%) TaRING-zf proteins were found at the cell nucleus. The location of RING-zf proteins is closely connected to their functions. In the nucleus, RING-zf proteins are primarily involved in the degradation of transcription factors and other nuclear expression proteins. The TaRING domains were divided into four canonical or modified types (RING-H2, -HC, -D, and -M). The RING-M was newly identified in *T. aestivum*, and might represent the intermediate states between RING-zf domain and other modified domains. The consensus sequence of the RING-M domain can be described as M-X_2_-R-X_14_-Cys-X_1_-H-X_2_-Cys-X_2_-Cys–X_10_-Cys–X_2_-Cys. The BRE, MBS, MeJA, and DRE-binding site were most frequently identified at *TaRING-zf* gene promoters. We detected 89, 66, 13, 57, 55, 56, and 1 gene pairs in *A. tauschii*, *B. distachyon*, *H. vulgare*, *O. Sativa*, *S. italica*, *Z. mays* as well as *A. thaliana*, respectively, suggesting that TaRING-zfs were closely related to genes of *Poaceae*. According to public RNA-seq data, in different developmental stages, 31.9% *TaRING-zf* genes were lowly expressed, 40.6% genes exhibited obviously high expression. Under abiotic stress treatment, 21% of 119 *TaRING-zf* genes were almost lowly expressed, 72.3% genes exhibited moderately or obviously strong expression. The 4 RING-HC (*TraesCS2A02G526800.1*, *TraesCS4A02G290600.1*, *TraesCS4B02G023600.1* and *TraesCS4D02G021200.1*) and 2 RING-H2 (*TraesCS3A02G288900.1* and *TraesCS4A02G174600.1*) were significantly expressed in 5 development stages, and they responded to drought stress. It makes them excellent candidates to create drought/heat-tolerence *T. aestivum* varieties. It could be of great help to use these genes to improve the quality and traits of *T. aestivum*. These findings could provide some helpful resources for future understanding the biological functions of *TaRING-zf* genes in wheat.

## Methods

### Plant materials and abiotic stress treatments

Bread wheat (*Triticum aestivum* L. cv. Fielder) materials were obtained from Prof. Xue Gangping’s lab in CSIRO Plant Industry [31]. *T. aestivum* seeds were germinated on wet filter paper at 4℃ for 5 days, and at 12℃ for 5 days. Seedlings were germinated in a greenhouse at 22℃. Three-leaf-stage seedlings were treated by heat, drought, and heat and drought stresses, and were treated at 0, 1, 6 h time points. The water-treated seedlings were used as mock controls. The abiotic stress treatments were performed in 37℃ incubator, 25% PEG6000 solutions, respectively. The plants growing normally (seedlings were germinated in a greenhouse at light 16 h/22℃, dark 8 h/16℃) and without any treatment of *T. aestivum* plants were used for measuring tissues specific expression patterns of 18 selected *TaRING-zf* genes [32]. The stress-treated plants were used for measuring different stresses response expression levels of 12 selected *TaRING-zf* genes.

### Identification and characterization of TaRING-zf genes

All *TaRING-zf* gene sequence information was acquired from and the Ensembl plants (http://plants.ensembl.org/index.html) to determine the features of TaRING-zf family members. Using the pfam (PF14634) (https://pfam.xfam.org/), the presence of a particular TaRING-zf domain (PF14634) in the resultant protein sequences was identified. Through the HMMER 3.0 tool, the obtained TaRING-zf domain was additionally searched for Hidden Markov Model (HMM) search with an E-value threshold of 1e-05 (Generally speaking, when E-value is less than 10^–5^, it indicates that the two sequences have high homology). The TaRING-zf domain was searched using the HMMER 3.0 tool for Hidden Markov Model (HMM) search with an E-value cutoff of 1e-05. Simple Modular Architecture Research Tool (SMART) (http://smart.embl-heidelberg.de/) was used to identify RING-domain and name for RING-variant domain. The ExPASy site (http://www.expasy.org/) was used to get data on the theoretical isoelectric point (pI), molecular weight (MW), atomic composition, instability index, aliphatic index, and total average hydrophilicity (GRAVY). TaRING-zfs subcellular localization data were obtained from the Cell-PLoc 2.0 server (http://www.csbio.sjtu.edu.cn/bioinf/Cell-PLoc-2/) [32].

### Cis-Elements analysis of TaRING-zf genes

The Ensemble Plants (http://plants.ensembl.org/index.html) database was used to obtain 1.5 kb DNA sequences from the TaRING-zf genes upstream of the start codon (ATG). To anticipate cis-regulatory elements in promoter regions, the PLACE database (https://www.dna.affrc.go.jp/PLACE/?action=newplace/) was employed [33].

### Chromosomal locations, gene duplication, and synteny analysis of TaRING-zf genes

The chromosomal position of all *TaRING-zf* genes was retrieved from Ensembl plants database. TBtool software was used to map the *TaRING-zf* genes onto chromosomes from chrA, chrB, and chrD, in ascending order of physical position (bps) [33].

The gene duplication occurrences were investigated using the Multiple Collinearity Scan toolbox (MCScanX). The TBtool software was used to find synteny areas between TaRING-zf genes, as well as collinear blocks of TaRING-zf genes with 1 dicot (*Arabidopsis thaliana*—*A. thaliana*) and 6 monocots (*Zea mays*-*Z. mays*, *Hordeum vulgare*-*H. vulgare*, *Aegilops tauschii*-*A. tauschii*, *Brachypodium distachyon*-*B. distachyon*, *Oryza sativa Japonica*-rice, *Setaria italic*-*S. italic*). The TBtool was used to visualize TaRING-zfs gene duplication occurrences as well as synteny links amongst the aforementioned species [34]. Ka/Ks Calculator software was used to compute synonymous (Ks) and non-synonymous (Ka) ratios. Following that, the divergence time of collinear gene pairs was determined using the T = Ks/(2λ × 10^–6^) Mya (λ = 6.5 × 10^–9^)] approach [35].

### Phylogeny, gene structure construction, and motif analysis

The evolutionary relationship between TaRING-zfs was discovered by gathering TaRING-zf domain sequences from the Ensembl plants database. The TaRING-zf domain sequences were aligned using the neighbor-joining (NJ) approach and 1000 replications for the bootstrap test using the ClustalW alignment algorithm in MEGA7.0 software [32]. The TaRING-zfs exon–intron structure and motif were determined to use the Gene Structure Display Server (GSDS 2.0) (http://gsds.cbi.pku.edu.cn) and the Multiple Em for Motif Elicitation (MEME) suite Version 5.1.1 online application (https://meme-suite.org/meme/tools/meme). The following MEME settings were optimized: maximum number of motifs, 15; minimum motif width, 4; maximum motif width, 50 [32].

### Expression patterns and qRT-PCR analysis of TaRING-zf genes

To investigate the expression patterns of TaRING-zf genes in various tissues (roots, stems, leaves, spikes, and grains), developmental stages, and stress responses. The RNA-Seq data used for this study are acquired from the National Center for Biotechnology Information Short Read Archive (http://www.ncbi.nlm.nih.gov/sra/) and wheat expression database (http://www.wheat-expression.com/). The TPM (transcripts per kilobase million) value of each *TaRING-zf* gene was calculated [36]. 138 *TaRING-zf* genes were analyzed at 15 developmental stages and were shown on the bottom side: SR, RTLS, RMS, S1S, STNS, SATS, SL, LTTS, L2DAAs, SPTNS, SPMS, SPAS, G2DAAs, G14DAAs, G30DAAs [32]. 119 TaC3HC4-RING finger genes were analyzed under 6 different stages treatment: D1h, D6h, H1h, H6h, DH1h, DH6h [32].

Based on the manufacturer’s instructions. The total RNAs from different tissues and differently treated materials under heat, drought, and heat and drought stress were extracted using Trizol reagent (Invitrogen). RNA integrity and quality were detected by NanoDrop 1000 and electrophoresis. First-strand cDNA was synthesized by the SuperScript II Reverse Transcriptase (Invitrogen). qRT-PCR research revealed 18 tissue-specific TaRING-zf gene expression profiles and 12 TaRING-zf genes that respond strongly to heat and drought conditions. Table S5 lists the qRT-PCR primers. As an internal control, TaRP15 was employed. The qRT-PCR was done using the QuantiFast® SYBR® Green PCR kit according to the manufacturer's instructions, with three replications for each sample. 2^−ΔΔCt^ value [ΔΔCt = (CT_target/Cd_-CT_actin/Cd_- (CT_target/control_-CT_actin/control_)] was used to calculate the expression levels [37].

## Supplementary information

**Additional file 1: Figure S1.** The alignment of 138 TaRING-zf protein sequences.

**Additional file 2: Figure S2.** Phylogenetic relationships, gene structure and architecture of conserved protein motifs in 138 TaRING-zfs from ***T. aestivum.*** **a** The name of 138 TaRING-zf genes. **b** Exon, intron, and UTR structure of 138 *TaRING-zf* genes. Yellow boxes indicate untranslated 5’ and 3’ regions, green boxes indicate exon, and black lines indicate introns. The gene full length and protein length can be estimated by using the scale at the bottom. **c** The motif composition of TaRING-zf proteins. The motifs, numbers 1-15, are displayed in different colored boxes.

**Additional file 3: Figure S3. **Domain-based classification of *T. aestivum* RING-zf proteins. **a** Additional TM domain contained in the RING-zf protein. **b** Additional IBR domain contained in the RING-zf protein. **c** Additional coiled coil domain contained in the RING-zf protein. **d** Additional RPT1 domain contained in the RING-zf protein. **e** Additional RING-ZnF-CHY, RING-Zinc_ribbon_6 and pfam Lon-substr-bdg domains contained in the RING-zf protein. **f** Additional DEXDc, HIRAN and HELICc domains contained in the RING-zf protein. **g** Additional WD40 domain contained in the RING-zf protein. **h** 2 TaRING-zf proteins were only contained 2 or 3 RING-zf domains. The additional domains architecture was predicted by on-line SMART program (http://smart.embl-heidelberg.de/).

**Additional file 4: Table S1.** Detailed information of all 138 TaRING zinc finger genes identified in tomato genome.

**Additional file 5: Table S2.** One-to-one orthologous relationships among *T.aestivum* and *T.aestivum*, *A.thaliana*, *A. tauschii*, *B. distachyon*, *H. vulgare*, rice, *S.italica* and *Zea mays*, respectively.

**Additional file 6: Table S3.** The information of *cis*-acting elements on the promoters of *T. aestivum* RING-zf genes.

**Additional file 7: Table S4.** The information of transcriptome data of *T. aestivum* RING-zf genes.

**Additional file 8: Table S5.** The list of primers.

## Data Availability

All information of TaRING-zf sequences were acquired from and the Ensembl plants (http://plants.ensembl.org/Triticum_aestivum/Info/Index), the presence of specific TaRING-zf domain (PF14634) in the resulting protein sequences was determined using the pfam (PF14634) (https://pfam.xfam.org/), the simple Modular Architecture Research Tool (SMART) (http://smart.embl-heidelberg.de/) was used to identify RING-domain and name of RING-variant domain, the pI, MW and GRAVY datas were acquired from the ExPASy server (http://www.expasy.org/), the subcellular localization results of TaRING-zfs were queried using the Cell-PLoc 2.0 server (http://www.csbio.sjtu.edu.cn/bioinf/Cell-PLoc-2/). the *cis*-regulatory elements from the PLACE database (https://www.dna.affrc.go.jp/PLACE/?action=newplace/). The RNA-Seq data used for this study are acquired from the National Center for Biotechnology Information Short Read Archive (http://www.ncbi.nlm.nih.gov/sra/) and wheat expression database (http://www.wheat-expression.com/) under accession number SRP045409.

## Abbreviations

RING-zf: RING (Really Interesting New Gene) zinc finger
*T. aestivum*: *Triticum aestivum* L.
*Arabidopsis*: *Arabidopsis thaliana*
B. rapa: *Brassica rapa*
Tomato: *Solanum lycopersicum*
*O. tauri*: *Ostreococus tauri*
*Z. mays*: *Zea mays*
*H. vulgare*: *Hordeum vulgare*
*A. tauschii*: *Aegilops tauschii*
*B. distachyon*: *Brachypodium distachyon*
*Rice*: *Oryza sativa Japonica*
*S. italic*: *Setaria italic*
GFF3: Genome Annotation File
Chr: Chromosomes
ABA: Abscisic Acid
ARE: Anaerobic Inducible Element
MBS: MYB drought inducible binding site
DRE: DREB/CBF transcription factor recognition site
LTR: Low-Temperature Response Elements
TGA-element: Auxin Response Elements
MeJA: Methyl Jasmonate
ABRE: ABA Response Element
expVIP: Wheat Expression Browser
HMM: Hidden Markov Model
SMART: Simple Modular Architecture Research Tool
pI: Theoretical Isoelectric Point
MW: Molecular Weight
GRAVY: Total Average Hydrophilicity
MCScanX: Multiple Collinearity Scan toolkit
Ks: Synonymous
Ka: Non-Synonymous
NJ: Neighbor-Joining
GSDS: Gene Structure Display Server
MEME: Multiple Em for Motif Elicitation
