# Supplementary figures and images for: Genome-wide identification, new classification, expression analysis and screening of drought & heat resistance related candidates in the RING zinc finger gene family of bread wheat (Triticum aestivum L.)
Source: BMC Genomics. 2022 Oct 7;23:696. doi: 10.1186/s12864-022-08905-x (PMC9547421; doi:10.1186/s12864-022-08905-x)

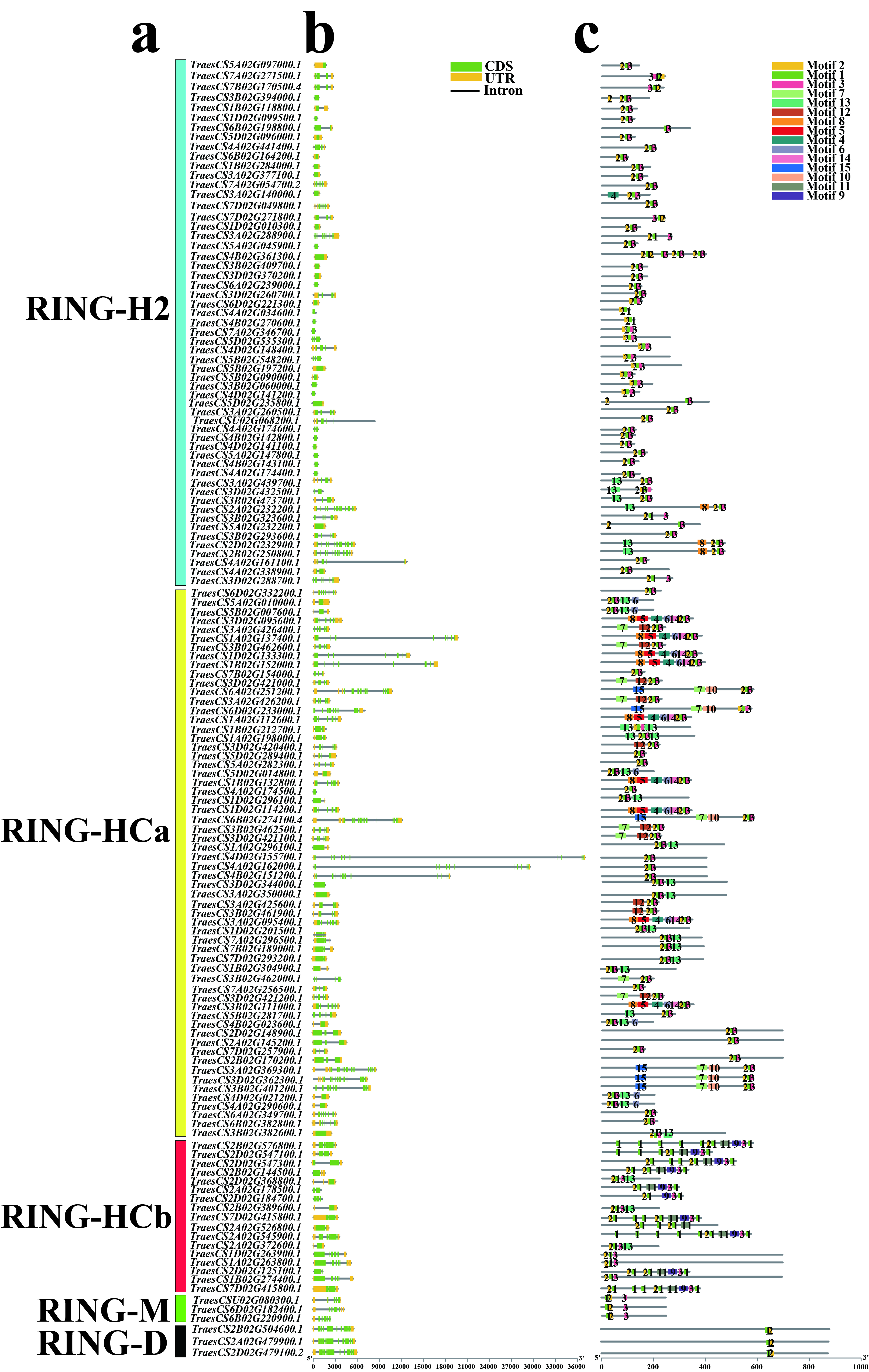

Supplement: Supplementary file 2 — Additional file 2: Figure S2. Phylogenetic relationships, gene structure and architecture of conserved protein motifs in 138 TaRING-zfs from T. aestivum. a The name of 138 TaRING-zf genes. b Exon, intron, and UTR structure of 138 TaRING-zf genes. Yellow boxes indicate untranslated 5’ and 3’ regions, green boxes indicate exon, and black lines indicate introns. The gene full length and protein length can be estimated by using the scale at the bottom. c The motif composition of TaRING-zf proteins. The motifs, numbers 1-15, are displayed in different colored boxes. [file 12864_2022_8905_MOESM2_ESM.jpg]

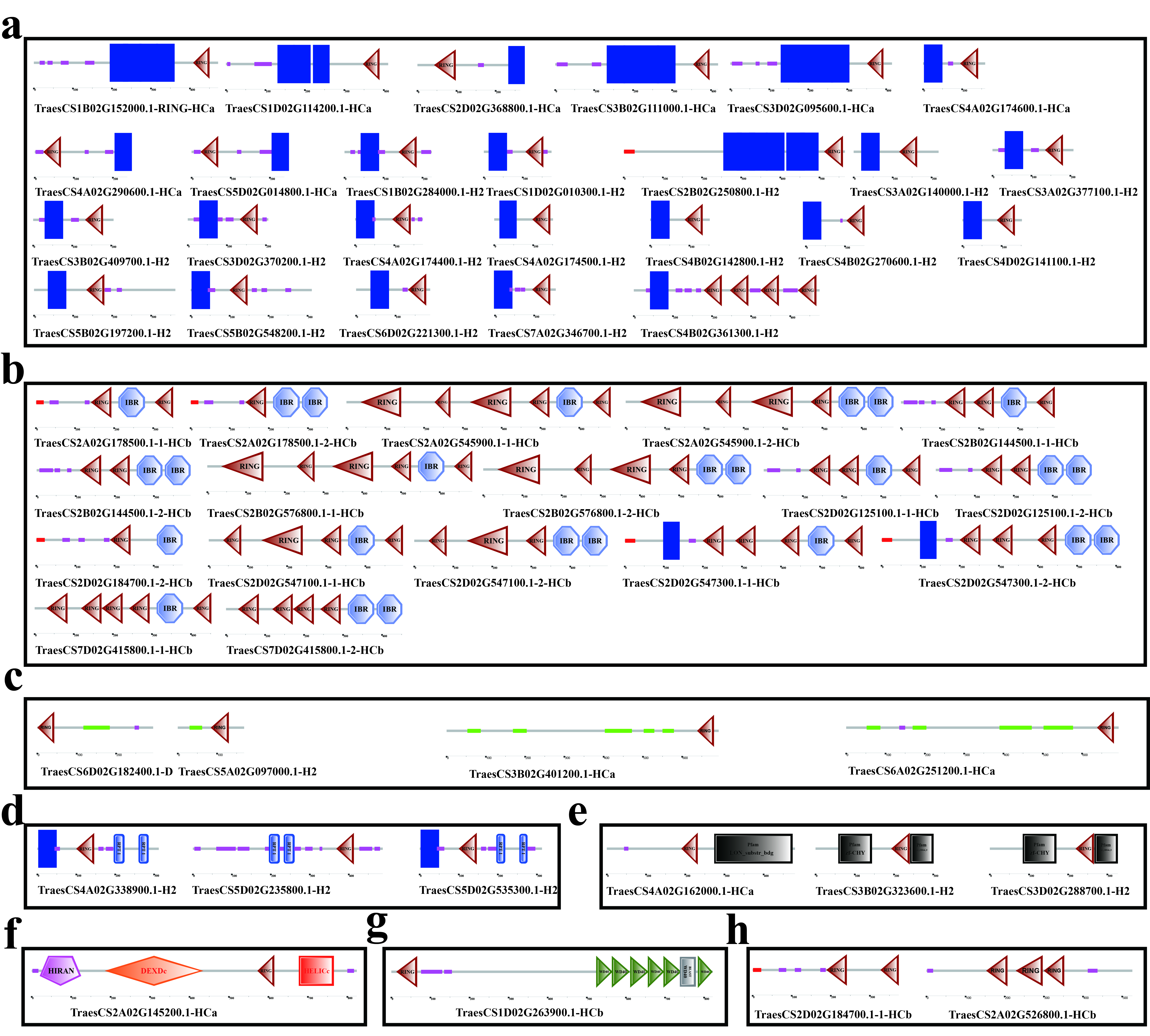

Supplement: Supplementary file 3 — Additional file 3: Figure S3. Domain-based classification of T. aestivum RING-zf proteins. a Additional TM domain contained in the RING-zf protein. b Additional IBR domain contained in the RING-zf protein. c Additional coiled coil domain contained in the RING-zf protein. d Additional RPT1 domain contained in the RING-zf protein. e Additional RING-ZnF-CHY, RING-Zinc_ribbon_6 and pfam Lon-substr-bdg domains contained in the RING-zf protein. f Additional DEXDc, HIRAN and HELICc domains contained in the RING-zf protein. g Additional WD40 domain contained in the RING-zf protein. h 2 TaRING-zf proteins were only contained 2 or 3 RING-zf domains. The additional domains architecture was predicted by on-line SMART program (http://smart.embl-heidelberg.de/). [file 12864_2022_8905_MOESM3_ESM.jpg]
